# MicroRNA Expression Patterns and Function in Endodermal Differentiation of Human Embryonic Stem Cells

**DOI:** 10.1371/journal.pone.0003726

**Published:** 2008-11-18

**Authors:** Galit Tzur, Asaf Levy, Eti Meiri, Omer Barad, Yael Spector, Zvi Bentwich, Lina Mizrahi, Mark Katzenellenbogen, Etti Ben-Shushan, Benjamin E. Reubinoff, Eithan Galun

**Affiliations:** 1 Goldyne Savad Institute of Gene Therapy, Hadassah University Hospital, Jerusalem, Israel; 2 Rosetta Genomics, Rehovot, Israel; University of Florida, United States of America

## Abstract

**Background/Aims:**

microRNAs (miRNAs) are small noncoding RNAs that regulate cognate mRNAs post-transcriptionally. Human embryonic stem cells (hESC), which exhibit the characteristics of pluripotency and self-renewal, may serve as a model to study the role of miRNAs in early human development. We aimed to determine whether endodermally-differentiated hESC demonstrate a unique miRNA expression pattern, and whether overexpression of endoderm-specific miRNA may affect hESC differentiation.

**Methods:**

miRNA expression was profiled in undifferentiated and NaButyrate-induced differentiated hESC of two lines, using microarray and quantitative RT-PCR. Then, the effect of lentiviral-based overexpression of liver-specific miR-122 on hESC differentiation was analyzed, using genomewide gene microarrays.

**Results:**

The miRNA profiling revealed expression of three novel miRNAs in undifferentiated and differentiated hESC. Upon NaButyrate induction, two of the most upregulated miRNAs common to both cell lines were miR-24 and miR-10a, whose target genes have been shown to inhibit endodermal differentiation. Furthermore, induction of several liver-enriched miRNAs, including miR-122 and miR-192, was observed in parallel to induction of endodermal gene expression. Stable overexpression of miR-122 in hESC was unable to direct spontaneous differentiation towards a clear endodermal fate, but rather, delayed general differentiation of these cells.

**Conclusions:**

Our results demonstrate that expression of specific miRNAs correlates with that of specific genes upon differentiation, and highlight the potential role of miRNAs in endodermal differentiation of hESC.

## Introduction

microRNAs (miRNAs) are endogenous ∼22-nucleotide non-coding RNAs, known to regulate the expression of target genes by at least two mechanisms–degradation of target mRNA transcripts [1]–[4] and inhibition of mRNA translation [5]. More than 700 human miRNAs have been identified so far according to miRBase release 10.0 (http://microrna.sanger.ac.uk/sequences/). Both the biogenesis and action of miRNAs rely on components of the RNA interference machinery, with several distinctions (for a comprehensive review see [6]). Systematic analysis of the spatial expression of miRNAs has shown that many miRNAs are expressed in a tissue-specific manner [7], [8]. While the functions and target genes of most miRNAs are still unknown, miRNAs have been engaged in many different functions, including developmental timing, patterning and embryogenesis, differentiation and organogenesis, growth control and apoptosis and may also be required for stem cell maintenance (reviewed in [9]).

Human embryonic stem cells (hESC) are derived from the inner cell mass of the human blastocyst, and are characterized by pluripotency and self renewal [10], [11]. Thus, hESC may serve as a model of early human embryology, and provide insights into human developmental processes [12].

Characterizations of miRNA expression in mouse [13]–[16] and in human [17]–[20] ES cells and ESC-derived embryoid bodies have been recently published, and revealed two highly-expressed clusters (miR-302 and mmu-miR-290/hsa-miR-371/372/373). Furthermore, specific miRNAs were proposed to modulate differentiation of mouse ES cells in recent studies [21], [22], but the role of miRNAs in the regulation of stem cell growth and differentiation is poorly understood. Likewise, data on miRNA function in human ES cell differentiation is particularly scarce. miRNA expression has been characterized in hESC-derived embryoid bodies, containing cells of all three germ layers (endoderm, ectoderm and mesoderm). However, characterization of miRNA expression in models of directed hESC differentiation has been published only for one protocol of differentiation towards extraembryonic endoderm [20], and a potential role for miRNAs in hESC differentiation has not yet been investigated.

In the present work, we performed a genome-wide analysis of miRNA expression in undifferentiated hESC and in hESC differentiated in the presence of NaButyrate (NaB), which was shown to promote endodermal differentiation [23], [24]. We further analyzed the effect of over-expression of the endoderm-specific miR-122 on spontaneous differentiation of hESC.

## Materials and Methods

### ES Cell Culture and Differentiation

Human ES cell lines, HES-1 and HES-2 [10], were cultured on human feeders (foreskin) in 85% Knockout (KO) DMEM medium supplemented with 15% KO-serum replacement, 1 mM L-glutamine, 50 U/mL penicillin, 50 µg/mL streptomycin, 1% nonessential amino acids (Gibco-BRL, Gaithersburg, MD) and 4 ng/mL basic fibroblast growth factor (bFGF, Cytolab, Rehovot, Israel) as described [25]. These ES cell lines were passaged every 6–7 days using 1 mg/mL type IV collagenase (Gibco). For the experiments we used cells from passages 18–40, which maintained a normal karyotype throughout the experiments. Both lines expressed the hESC markers, Oct4, SSEA-4 and Tra-1-81, in at least 80% of the cells, as determined by flow cytometry analysis (data not shown). For differentiation, cells were transferred from feeders to human fibronectin-coated dishes (12.5 µg/cm^2^, BD Biosciences, Bedford, MA) and grown in the same culture medium that was conditioned with the feeder cells for 24 hr prior to use. After three days, the medium was replaced with unconditioned medium without bFGF, supplemented with 0.5 mM NaButyrate (Sigma, St. Louis, MO) for directed differentiation, or without NaB for control spontaneous differentiation. Cells were harvested after seven or fourteen days with EDTA (Gibco) for total RNA isolation. Neural spheres were derived from HES1 cells as described [26].

### RNA Isolation

Total RNA was isolated from cells or tissues (human liver tissues were obtained under IRB approval) with Trizol reagent (Invitrogen, Carlsbad, CA) according to the manufacturer's instructions, except that RNA precipitation was performed with ethanol.

### MicroRNA Microarray

MicroRNA expression analysis was performed as previously described [27]. Briefly, cRNA was derived from adaptor-ligated to 100 µg size-fractionated RNA from each sample. Following amplification, the double-stranded cDNA, carrying a T7 RNA polymerase promoter on the 3′ adaptor, was used for the labeling reaction. Labeled cRNA (lcRNA) incorporating either Cy3 or Cy5 was purified through a G-50 column and hybridized under standard conditions with custom microarrays by Agilent Technologies. The custom array design was as described [28], including oligonucleotides matched to validated human microRNA (Sanger Rfam registry) and thousands of Rosetta Genomics predicted microRNAs. Raw signals vary from a minimal signal of ∼50 to a saturated signal of ∼50,000. All probes were directed against human miRNAs, and the prefix ‘hsa’ was either present or not in a miRNA name only for the sake of convenience. Expression data from the microarray was normalized using polynomial fitting. First, the mean expression level for each miRNA in all experiments was calculated, and then a 2^nd^ degree polynomial function F for fitting each experiment signals to the calculated mean expression was found. The normalized signal for each miRNA in each experiment is the result of F(x), where F is the specific function for the specific experiment. Only validated miRNAs (published and predictions of Rosetta Genomics, designated as MIDxxx) probes were used for the normalization (the minority of probes).

### microRNA cloning and sequencing

Few of Rosetta predicted microRNAs that had high expression on the microarrays were cloned as previously described [28]. Briefly, biotinylated capture oligonucleotides (22–30 nucleotides long, with biotin at the 5′ end) were hybridized to an aliquot (5 µl) of the library in TEN buffer. mMACS Streptavidin Microbeads were then added and the reaction was incubated for 2 min at the hybridization temperature. Mixture was loaded onto a magnetized mMACS Streptavidin Kit column and hybridized single-stranded library molecules were eluted by adding 150 µl of water preheated to 80°C. The single-stranded cDNA library molecules were recovered, amplified by PCR, ligated into a pTZ57R/T vector and transformed into JM109 bacteria. Positive colonies were identified and sequenced.

### cDNA Macroarray

cDNA macroarrays containing probes for 83 genes (including undifferentiated hESC and hepatic markers and endogenous controls) were fabricated as described [29]. Briefly, cDNA probes for 83 genes were amplified by RT-PCR, cloned into TOPO-TA vector (Invitrogen), sequence-verified, and spotted on nylon membranes (GeneScreen™, NEN, Boston, MA). 5 µg of total RNA from each sample were labeled with dCTPαP^33^, and hybridized with the membrane as described [29]. Gene expression was quantified using VisualGrid™ software and the Matlab™ program “MembraneProcess” [29]. Average results of two independent experiments were used.

### Algorithm for Prediction of Regulation of miRNAs by Transcription Factors

In order to predict regulation of miRNAs by transcription factors the following algorithm was used. First, a binary value was declared for each transcription factor (TF) in each sample as expressed/unexpressed, based on the results of the cDNA macroarray (background threshold was considered as 0.01 for this analysis). Then, a binary value was declared for each miRNA in each sample as expressed/unexpressed, based on the results of the miRNA microarray (background threshold was variable and estimated manually for each sample). For each TF, a list of miRNAs which were co-expressed with the TF in all the samples was compiled. For example, if TF A was expressed in sample 1 but not in the other samples, then all the miRNAs which were expressed in sample 1 and not expressed in the other samples were considered as co-expressed with this TF. In these cases, the TF was predicted to serve as an activator. In cases of anti-correlation between the TF expression and the miRNA expression, the TF was predicted to serve as a repressor. For prediction of TF binding to miRNA promoter, a transcription start site (TSS) was predicted for all miRNAs using annotated genes, mRNAs, ESTs, Paired End Tags (PET) sequences, and CpG islands downloaded from the UCSC genome browser (http://genome.ucsc.edu/). Promoter was predicted similarly to what was described by [30]. All predicted transcription factor binding sites taken from the UCSC genome browser (TFBS track representing Transfac data) were searched in a genomic area of 2000 nucleotides (nts) upstream and 2000 nts downstream to the TSS, to yield a dataset of all miRNAs and the TFs capable of binding to their promoters (unpublished dataset). Lastly, for each co-expressed or anti-correlated miRNA/TF pair, we tested whether the TF could directly bind to the miRNA promoter according to the above dataset. TF/miRNA pairs, in which the miRNA was derived from a poly-cistronic miRNA cluster, were included only in the case that all miRNAs derived from this cluster were co-expressed or anti-correlated with the TF.

### Vector Design and Virus Production

Construction of a lentiviral vector expressing enhanced yellow fluorescent protein (EYFP) reporter fused to the 3′UTR of miR-122 target gene CAT1 [31] was performed as follows: First, the human PGK promoter-EGFP cassette from pRLLSIN18.hPGK.EGFP [32], [33] was inserted into *ECO*RV and *Bsp*1407I sites of pSIN18.cPPT.hEF1αp.EGFP.WPRE [34]. Then, by digestion of the resulted plasmid with *Bsp*1407I and *Bam*HI, the EGFP cassette was replaced with a polylinker containing *Hpa*I site, to generate pSIN18.cPPT.hPGKp.linker.WPRE. Finally, an *Eco*47III and *Hpa*I fragment of EYFP-CAT1 cassette derived from plasmid CAT1 in pEYFP Stop was inserted into *Hpa*I site of pSIN18.cPPT.hPGKp.linker.WPRE to generate pSIN18.cPPT.hPGKp.EYFP-CAT1.WPRE.

For construction of a lentiviral vector expressing miR-122, the human H1 promoter (position -220 to +1) was amplified by PCR and subcloned into pBS as described [35]. The promoter was then digested with *Xho*I and *ECO*RV and inserted into viral vector pSIN18.cPPT.hEF1αp.RFP.WPRE [35] to generate pSIN18.cPPT.H1p.hEF1αp.RFP.WPRE. The genomic sequence encoding hsa-miR-122 (a 277-base fragment) was amplified by PCR from plasmid 122 in pCMV using primers: 5′-AACCATCGATATGCTTCTTTTCTCTGCTTAGG (*Cla*I), and 5′-AAAAGTACTAAAAACAAGATTGAGAAGACTGATATC (*Sca*I). The amplified fragment was cloned into the *Cla*I and *ECO*RV sites of pSIN18.cPPT.H1p.hEF1αp.RFP.WPRE to generate pSIN18.cPPT.H1p.miR-122.hEF1αp.RFP.WPRE. For generation of mutated miR-122-expressing vector, three mutations in the seed sequence (at positions 2, 4 and 6) were introduced by PCR. The sequence of the miRNA was amplified from pSIN18.cPPT.H1p.miR-122.hEF1αp.RFP.WPRE by PCR using primers: 5′-GAGGTGAAGTTAACACCTTCGTGGCTACAGAGTTTCCTTAGCAGAGCTGTCGTGAGTGACAATGGTGTTT (*Hpa*I), and 5′-GATTGAGAAGACTGATATCAGATGAACCTT (*ECO*RV) and recloned into sites *Hpa*I and *ECO*RV of this vector. The basic vector pSIN18.cPPT.H1p.hEF1αp.RFP.WPRE served as an empty vector. All vectors were sequence-verified. Recombinant virions of miR-122-expressing vectors were produced and concentrated as previously described [34].

### Transduction of hESC

At the time of routine passage, hESC were dissociated into a single cell suspension by EDTA (Gibco) and plated in a feeder-covered 6-well tissue culture plate at a concentration of 1×10^5^ per well. The next day, the medium was replaced and supplemented with the concentrated virus, in the presence of 5 µg/ml polybrene (Sigma). The medium was replaced with fresh hESC culture medium one day following transduction.

### DNA Microarrays

Gene expression profiling was performed using the Affymetrix Human Genome U133A 2.0 Array (Santa Clara, CA), comprised of ∼22,000 probe sets representing 18,400 transcripts and variants, including 14,500 well-characterized human genes, according to the manufacturer's instructions. Data was normalized using RMA software (R2.1.0 package) and analyzed using SpotfireDecisionSite™ software for functional genomics. For analysis of gene expression in RNA from differentiated hESC expressing miR-122, average results of three (mutant miRNA) or two (wt miRNA) independent experiments were used. The datasets have been deposited in NCBI's Gene Expression Omnibus [36] and are accessible through GEO Series accession number GSE13460 (<http://www.ncbi.nlm.nih.gov/geo/query/acc.cgi?acc=GSE13460>).

### Flow Cytometry

Cells were harvested for fluorescence-activated cell sorter (FACS) analysis with EDTA (hESC) or 0.25% Trypsin/1 mM EDTA (HEK-293 cells) (both from Gibco) , and suspended in phosphate–buffered saline containing 2% fetal calf serum and 0.1% sodium azide. The cells were analyzed on a FACSCalibur system (Becton-Dickinson, San Jose, CA) using the CellQuest™ software.

### Real-time Quantitative Polymerase Chain Reaction

For mature microRNA quantification, a two-step protocol including reverse transcription with a miRNA-specific primer and TaqMan MicroRNA Reverse Transcription Kit, followed by real-time PCR with TaqMan assays (human) and Taqman PCR Master Mix Kit, was applied on ten nanograms of total RNA for each sample (all reagents were purchased from Applied Biosystems [ABI], Foster City, CA), and reaction protocols were carried out according to the manufacturer's instructions. For mRNA quantification, 2.5 µg of total RNA were reverse transcribed using random hexamer primer and Moloney murine leukemia virus reverse transcriptase RNase H minus (both from Promega, Madison, WI). cDNA was amplified with Fast Taqman PCR Master Mix Kit and inventoried human Taqman assays (both from ABI), according to the manufacturer's instructions.

The reactions for miRNA and mRNA were automated by a 7900HT Fast Real-Time PCR System (ABI). Each PCR reaction was performed in triplicate and the average ct was used for RQ calculation after normalization to RNU43 (for miRNAs) and human GUSB (for mRNAs) (both from ABI).

### Statistical Analysis

Data is expressed using the mean and the standard deviation when at least two independent experiments were performed. Student's *t* test was used for performing analysis of variance in Excel software. A *p* value of 0.05 or less was considered statistically significant.

## Results and Discussion

### Characterization of miRNA expression in undifferentiated hESC

**Figure 1:**
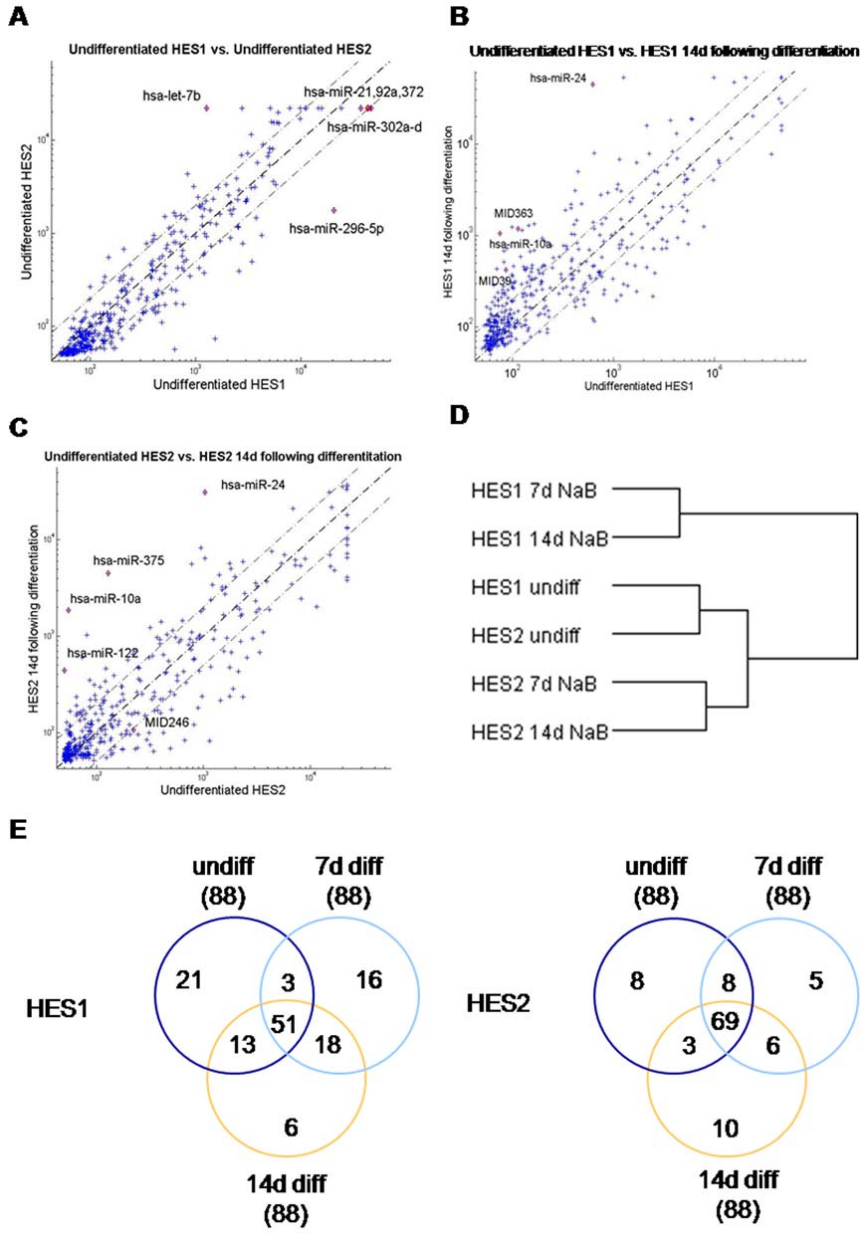
miRNA expression analysis in undifferentiated and differentiated hESC. A–C. Scatter plots representing miRNA expression profiles of 436 miRNAs in undifferentiated and 14d-differentiated HES1 and HES2 cells. Names of outlier miRNAs are indicated. miRNA names in the format MIDxxx are predictions of Rosetta Genomics. D. Unsupervised hierarchical cluster analysis was performed on expression signature of 436 human miRNAs in undifferentiated (undiff) HES1 and HES2 cells, and in cells differentiated (diff) with NaB for 7d or 14d (Cluster 3.0 software, log transformed data, average linkage). A dendogram demonstrating similarity level in miRNA expression between the various samples is shown. E. Venn diagrams of 20% of the miRNAs with the highest expression level for each cell line in undifferentiated (undiff), 7d-differentiated (7d diff) and 14d-differentiated (14d diff) HES1 and HES2 cells. Number of miRNAs included in each circle is denoted in parentheses.

We first characterized the miRNA expression profile of our undifferentiated hESC lines and compared it with published data. To this end, we extracted RNA from two different hESC lines (HES1 and HES2 [10]) and subjected it to miRNA expression profiling of 436 human miRNAs using specialized miRNA microarrays [28] (Table S1). During the miRNA profiling we discovered three novel miRNAs, one of which was first cloned in undifferentiated HES2 cells and whose expression level changed upon differentiation (Table 1). The expression level of these novel miRNAs was not consistent in our two cells lines, and additional experiments are needed in order to clarify whether these miRNAs play a role in hESC differentiation. Examination of the miRNA expression profiles of HES1 versus HES2 cells (Fig. 1A) revealed miRNAs that were highly expressed in both lines, such as miR-21, miR-372 and members of the cluster 302. Table 2 lists the miRNAs with the highest expression levels for each cell line. 82% of these miRNAs were highly-expressed in both lines. These results are consistent with previous reports [13], [17], and show that undifferentiated hESC express a unique set of miRNAs, including two ESC clusters on chromosomes 4 (cluster 302) and 19 (cluster 371-3). We also detected high expression of members of the nonconserved cluster (cluster 520) as in [20]. Notably, several of the top expressed miRNAs (miR-93, cluster 520, cluster 302 and cluster 372-3) contain the same seed sequence and are thus predicted to target the same mRNAs (according to TargetScan 4.0). Co-expression of different miRNAs targeting the same genes in the same tissue may confer higher efficiency and flexibility to the miRNAs' action.

**Table 1:** Novel miRNAs identified in hESC

Name	Sequence a	Number of clones b	Cloning source library	Comments	Expression in hESC
MID246	ATTTGTGCTTGGCTCTGTCA(C)	2	Undifferentiated HES2	Derived from a highly conserved area. Very stable hairpin.	Downregulated only in HES2 in response to NaB
MID363	CTGTACAGCCTCCTAGCTTTCC	2	Brain-Substantia nigra	miR* of hsa-let-7a in hsa-let-7a-2	Upregulated only in HES1 in response to NaB
MID39	(A)AAATGGTGCCCTAGTGACTAC(A)	3	Placenta	miR* of hsa-miR-224. Cloned with variable 5′ and 3′ ends	Upregulated only in HES1 in response to NaB

a Nucleotides that were optional during sequencing are in parenthesis.

b of all variants.

**Table 2:** The top 10% miRNAs expressed in undifferentiated hESC

	HES1	HES2
1	**hsa-miR-21**	**hsa-miR-21**
2	**hsa-miR-302a**	**hsa-miR-302a**
3	**hsa-miR-302b**	**hsa-miR-302b**
4	**hsa-miR-302c**	**hsa-miR-302c**
5	**hsa-miR-372**	**hsa-miR-372**
6	**hsa-miR-92a**	**hsa-miR-302d**
7	**hsa-miR-302d**	**hsa-miR-125b**
8	**hsa-miR-125b**	**hsa-miR-221**
9	hsa-miR-296-5p	**hsa-miR-205**
10	**hsa-miR-221**	**hsa-miR-92b**
11	**hsa-miR-205**	**hsa-miR-25**
12	**hsa-miR-92b**	**hsa-miR-106a**
13	**hsa-miR-25**	hsa-let-7b
14	**hsa-miR-222**	**hsa-miR-30c**
15	**hsa-miR-15b**	**hsa-miR-92a**
16	**hsa-miR-106a**	**hsa-miR-222**
17	**hsa-miR-30d**	**hsa-miR-199a-5p**
18	**hsa-miR-27b**	**hsa-miR-27b**
19	**hsa-miR-191**	**hsa-miR-15b**
20	**hsa-miR-512-3p**	**hsa-miR-125a-5p**
21	**hsa-miR-17**	**hsa-miR-17**
22	**hsa-miR-517b**	**hsa-miR-191**
23	**hsa-miR-19b**	**hsa-miR-30d**
24	**hsa-miR-125a-5p**	**hsa-miR-93**
25	**hsa-miR-30c**	**hsa-miR-182**
26	**hsa-miR-505**	**hsa-miR-505**
27	**hsa-miR-331-3p**	**hsa-miR-19b**
28	**hsa-miR-26a**	**hsa-miR-26a**
29	**hsa-miR-151-3p**	**hsa-miR-148a**
30	**hsa-miR-182**	hsa-miR-30a
31	**hsa-miR-197**	**hsa-miR-151-3p**
32	hsa-miR-371-3p	**hsa-miR-339-5p**
33	**hsa-miR-339-5p**	**hsa-miR-331-3p**
34	hsa-miR-520f	hsa-miR-214
35	**hsa-miR-328**	**hsa-miR-197**
36	hsa-miR-142-3p	hsa-miR-200c
37	**hsa-miR-326**	**hsa-miR-517b**
38	**hsa-miR-93**	**hsa-miR-512-3p**
39	**hsa-miR-148a**	hsa-miR-345
40	hsa-miR-302c*	hsa-miR-199a-3p
41	hsa-miR-363	**hsa-miR-328**
42	hsa-miR-130b*	hsa-miR-30e
43	hsa-miR-373	**hsa-miR-326**
44	**hsa-miR-199a-5p**	hsa-miR-20b

The top 10% of miRNAs expressed at the highest level are shown for each cell line, in a descending order of expression. miRNAs that appear in both cell lines are **bolded**.

Interestingly, we detected statistically significant enrichment of oncomiR expression in our hESC lines, including members of the paralogues clusters 17–92, 106a-92 and 106b-25 (hypergeometric test, p<0.0005). Expression of proto-oncomiRs, engaged in regulation of cell cycle, may be expected in hESC, as hESC has an indefinite proliferation capacity.

### Effect of NaButyrate-induced differentiation of hESC on miRNA expression

The histone deacetylase inhibitor, NaButyrate (NaB) was shown to induce endodermal [23] and hepatic-like [24] differentiation of both hESC and embryonal carcinoma cells [36]. We sought to induce endodermal differentiation by a 7 and 14 day (d) treatment with NaB.

Cluster analysis of miRNA expression uncovered a distinct fingerprint for undifferentiated and NaB-differentiated hESC (Table S1 and Fig. 1B–D). Nonetheless it is worthwhile noting that there were cell-line specific profiles, similar to what was recently demonstrated by Melton and colleagues [37]. A substantial overlap in miRNA expression before and after NaB differentiation (Fig. 1E), including persistence of the ESC-specific cluster 302, may suggest that our protocol only partially pushes the cells towards differentiation.

Among the downregulated (Table 3) miRNAs were ESC-enriched miRNAs such as cluster 302 and miR-106a [13], [14], miR-17-5p [17] and miR-124 [13], [17]. In the adult, miR-124 is expressed specifically in the brain [7], and its downregulation may suggest that neural differentiation was probably not promoted by NaB treatment. miR-24 was predominantly upregulated in both lines (Table 3). A miR-24 validated target is Notch1 [38]. Notch-signaling was shown to inhibit endoderm formation in zebrafish [39], and hence, it is intriguing to consider miR-24 involvement in repression of Notch signaling as a component in promoting endodermal differentiation. miR-10a, yet another miRNA to be upregulated by NaB treatment in both lines, is upstream of HOXA1 [40]. HoxA1 was proposed to mediate repression of endodermal differentiation [41], which is consistent with higher expression of endodermal markers in Hoxa1^−/−^ mouse ES cells. Thus, induction of miR-10a and miR-24 in response to NaB may contribute to endodermal differentiation via HOXA1 and Notch repression.

**Table 3:** miRNAs upregulated and downregulated upon hESC differentiation

Upregulated miRNAs					
HES1			HES2		
miRNA ID	7d diff/undiff	14d diff/undiff	miRNA ID	7d diff/undiff	14d diff/undiff
hsa-miR-24	71.93	72.06	hsa-miR-375	13.09	34.76
hsa-let-7b	31.48	42.25	hsa-miR-10a	2.60	34.10
hsa-miR-10a	2.22	13.96	hsa-miR-24	14.98	30.29
hsa-let-7c	2.54	12.49	hsa-miR-218	3.06	12.67
hsa-miR-346	69.70	12.33	hsa-miR-122	2.05	8.81
hsa-let-7d*	89.86	11.25	hsa-miR-371-3p	12.44	8.74
MID363	10.25	10.45	hsa-miR-371-5p	10.49	8.11
hsa-miR-15a*	9.16	9.18	hsa-miR-373	14.96	7.26
hsa-miR-188-3p	2.51	8.98	hsa-miR-30b	1.41	6.26
hsa-miR-509-3p	110.20	8.25	hsa-miR-500*	2.15	4.01
**Downregulated miRNAs**					
hsa-miR-200c	0.08	0.09	hsa-let-7i	0.12	0.07
hsa-miR-107	0.09	0.14	hsa-miR-302c*	0.76	0.10
hsa-miR-17	0.05	0.15	hsa-miR-409-3p	0.18	0.12
hsa-miR-20b	0.04	0.15	hsa-miR-187	0.21	0.14
hsa-miR-106a	0.04	0.16	hsa-miR-142-3p	0.15	0.14
hsa-miR-301a	0.18	0.18	hsa-miR-520f	1.51	0.15
hsa-miR-423-5p	0.87	0.18	hsa-miR-654-3p	0.19	0.15
hsa-miR-124	0.14	0.19	hsa-miR-485-3p	0.15	0.15
hsa-miR-20a	0.22	0.20	hsa-miR-302d	1.16	0.18
hsa-miR-130a	0.12	0.20	hsa-miR-93	0.34	0.18

List of the top ten miRNAs upregulated or downregulated at least 2-folds in each line upon differentiation. Expression is presented as fold of change in 7d and 14d differentiated (diff) versus undifferentiated (undiff) cells. miRNAs designated as MIDxxx are predictions of Rosetta Genomics.

Let-7b/c/d* family members were also upregulated upon differentiation, an observation that nicely fits with the avoidance of let-7 activity in ESCs [42].

The expression of endoderm-specific miRNAs–miR-375 [43], and miR-122, [31] was upregulated in response to NaB, though to a higher extent in the HES2 cell line. Induction of these miRNAs may indicate an endodermally-oriented differentiation of our cells, as expected with NaB treatment.

Taken together, we suggest that miRNAs 10a, 24 and 122 mark endodermal differentiation. According to the data of Laurent et al., miR-122, miR-10a and miR-24 were upregulated in hESC differentiated towards extraembryonic endoderm, while miR-375's expression was unchanged [20]. When comparing these results to ours, it could be that miR-375's upregulation further allows for the distinction of definitive vs. extraembryonic endoderm.

Next, we compared the miRNA expression in our NaB-treated samples to that of adult liver. More specifically, we chose the most tissue-specific human miRNAs, which are relatively abundant in the liver, according to the miRNA atlas reported recently by Tuschl and colleagues [44]. We discovered that of these five miRNAs, miR-122 and miR-192 were upregulated at least 1.5-fold in both HES1 and HES2 cells, while miR-135b and miR-33a were upregulated only in HES2 cells, and miR-224 was upregulated only in HES1 cells.

**Figure 2:**
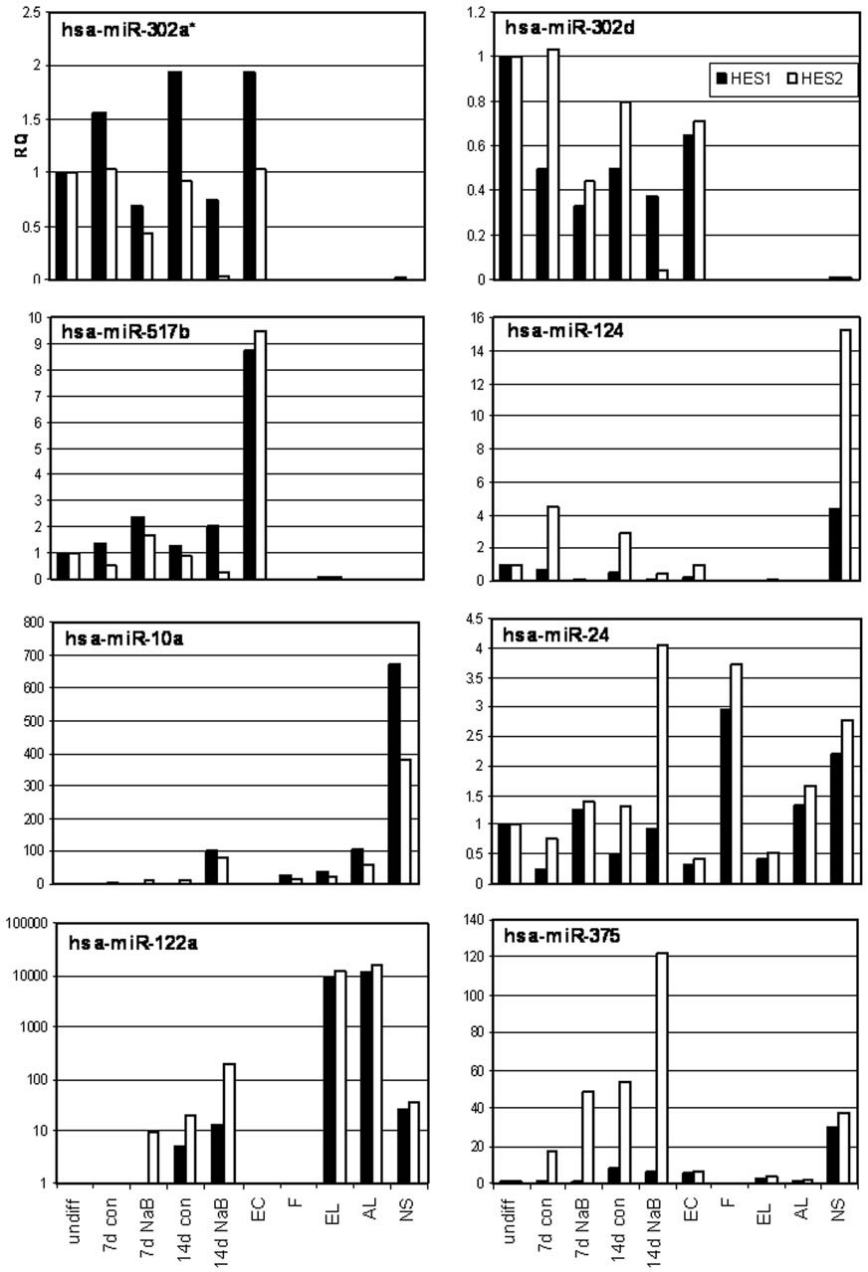
Verification of the miRNA microarray results by qRT-PCR. QRT-PCR results of miRNA expression level in undifferentiated HES1 and HES2 cells or cells differentiated with NaB for 7d or 14d. Results are expressed as relative quantification of the miRNA expression level in each sample relative to undifferentiated cells of the respective line, and normalized to RNU43. Each reaction was performed in triplicate and the average ct was used for RQ calculation. RQ–Relative Quantification; Con–control cells grown with the same media as treated cells, but without NaB; NaB–0.5 mM Na Butyrate; F–Feeder, mitotically arrested foreskin cells (feeder cells of hESC); EC–Embryonal Carcinoma 2102Ep cell line; EL–human embryonic liver of stage 7w from gestation; AL–human normal adult liver; NS–Neurospheres, HES1 cells differentiated for 4.5w towards neural fate.

The expression pattern of eight miRNAs was verified by quantitative (q) RT-PCR (Fig. 2). These miRNAs were differentially-expressed upon NaB-induced differentiation and represent ES miRNAs (hsa-miR-302a*, hsa-miR-302d, hsa-miR-517b), endodermal miRNAs (hsa-miR-122, hsa-miR-375) and miRNAs that were upregulated in both lines (hsa-miR-10a, hsa-miR-24). Generally, the RT-PCR results confirmed the microarray results, but the fold change was usually higher in the RT-PCR. Different expression of these miRNAs in feeder cells compared to hESC and hESC-derived cells showed that these expression patterns were not a result of feeder cell contamination.

**Figure 3:**
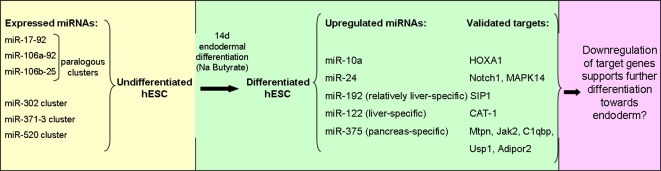
Summary of miRNA expression in endodermal differentiation of hESC.

A summary of miRNA expression in hESC differentiated with NaB is depicted in Figure 3. On the whole, many of the miRNAs that were either upregulated or downregulated upon differentiation are related to proliferation, an observation which is consistent with NaB's ability to affect cell cycle and proliferation [45]. Two of the most upregulated miRNAs common to both of our cell lines were miR-24 and miR-10a, whose target genes have been shown to inhibit endodermal differentiation. Additional miRNAs that are considered as relatively liver-specific or endoderm-specific, such as miR-122, miR-192 and miR-375 were also induced in both lines upon differentiation, albeit to a greater extent in HES2 cells. Additional experiments should clarify whether these miRNAs play a role in endodermal differentiation of hESC or in the embryo.

### Characterization of hepatic gene expression in hESC differentiated with NaB

**Figure 4:**
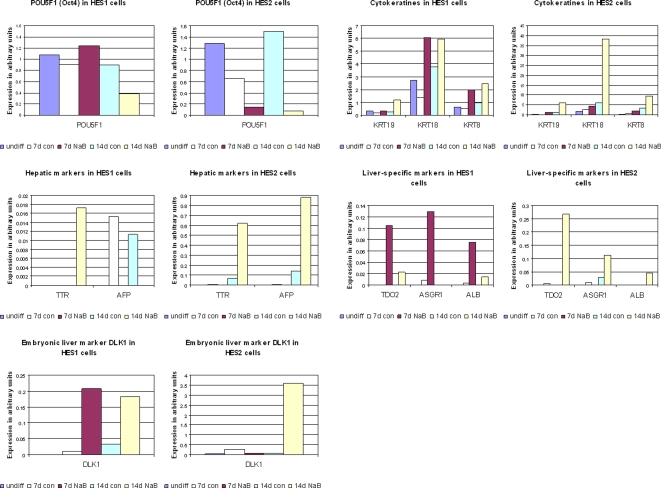
Characterization of hepatic markers expression in hESC differentiated with NaB. cDNA macroarray results of gene expression analysis in undifferentiated (undiff) HES1 and HES2 cells or cells treated with NaB for 7 or 14d, or with the basic medium only (con). The same RNA samples that were utilized for miRNA profiling were used for this analysis. The average results of two independent experiments are presented as gene expression level in arbitrary units. CK8-cytokeratin 8; CK18–cytokeratin 18; CK19–cytokeratin 19; AFP-alpha-fetoprotein; TTR-transthyretin; ALB–albumin; ASGR1-asialoglycoprotein receptor 1; TDO2-tryptophan 2,3 dioxygenase; DLK1-Delta-like 1 Homolog.

To evaluate the level and fate of hESC upon differentiation induced by NaB, we determined the expression profile of 83 hESC and liver markers using a homemade cDNA macroarray. Results of selected genes are presented in Figure 4. Oct4 (POU5F1), a marker for undifferentiated hESC, was downregulated in cells treated with NaB compared to undifferentiated cells. The downregulation was more prominent in HES2 than in HES1 cells, in accordance with the more prominent downregulation of ESC-specific miRNAs in differentiated HES2 cells. Cytokeratins are markers of epithelium and are expressed in hepatocytes (cytokeratine 8, 18) and cholangiocytes (cytokeratin 19), but are not specific to the liver. All three markers were expressed at very low levels in the undifferentiated cells, but were upregulated after treatment with NaB, which may indicate that the differentiated cells acquired an epithelial identity. Likewise, hepatic markers such as alpha-fetoprotein (AFP) and transthyretin (TTR), which are also expressed in extra-embryonic endoderm, were not expressed in undifferentiated cells and were upregulated in response to NaB, most prominently in HES2 cells after fourteen days. Hepatocyte-specific markers such as albumin (ALB), asialoglycoprotein receptor 1 (ASGPR1) and tryptophane 2, 3 dioxygenase (TDO2) were also not expressed in undifferentiated cells, and all three markers were upregulated in response to NaB. This upregulation was more significant in HES1 cells after seven days and in HES2 cells after fourteen days. Expression of the embryonic liver marker delta-like homolog 1 (DLK1) [46] was also upregulated in response to NaB, yet again, to a much greater extent in HES2 cells. In conclusion, the cells differentiated in response to NaB, as indicated by the downregulation of Oct4, and seemed to gain an epithelial identity and to express known liver markers. In response to NaB, HES2 cells expressed more hepatic markers and at higher levels compared to HES1 cells, in accordance with the differences in the level of endodermal miRNA expression between these lines. In addition, it seemed that for hepatic gene induction, fourteen days was the preferred time period of NaB treatment for HES2 cells, while a seven-day treatment was more favorable for HES1 cells.

These findings suggest that specific miRNA expression correlates with specific gene expression in differentiated cells, as was the case with undifferentiated hESC in the present study and in others [16], [18], [19].

### Prediction of transcription factors that affect miRNA expression in differentiated hESC

In an effort to uncover the regulatory factors (activators and repressors) responsible for the changes in miRNA expression upon hESC differentiation, we compared our gene expression data with the miRNA expression data. Towards this aim, we developed an algorithm which allowed us to correlate between differentially-expressed transcription factors (TFs) and miRNAs, and then for each given pair of miRNA/TF, to search the promoter region for a conserved binding site for the TF. The expression of eleven out of twenty-one TFs included in our cDNA macroarray changed by at least two-fold in at least one of our samples (not shown). Our analysis predicted two activators and two repressors, which could have affected our miRNA expression upon differentiation (Table 4). One TF, CEBPA (C/EBPα), was predicted to affect miRNA expression both positively and negatively, according to our results. CEBPA is highly-expressed in the developing and adult liver, as well as in additional metabolic tissues and the hematopoietic tissue, where it controls differentiation-dependent gene expression and inhibits cell proliferation ([47] and references therein). In line with CEBPA's known functions, our results predicted it to activate let-7c and miR-125b, both of which are capable of inhibiting cell proliferation [48], [49], and in addition, to repress miR-130a, which was shown to be involved in megakaryocytopoiesis [40]. Interestingly, CEBPA, which was upregulated four-fold in our HES1 cells upon differentiation, is predicted to be itself regulated by miR-124, miR-25, miR-363 and miR-367 (among others) according to TragerScan 4.2, all of which were downregulated at least 1.5-fold in differentiated HES1 cells. Collectively, this data enhances our understanding of regulation of hESC differentiation by miRNAs, yet necessitates further support in additional targeted experiments.

**Table 4:** Transcription factors predicted to regulate miRNA expression in differentiated hESC

Transcription factor	Regulated miRNA	miRNA chromosomal location	Strand	Predicted TSS	miRNA precursors in the transcript	Location of TFBS relative to TSS	Z Score^a^
**Predicted activators:**							
CEBPA	hsa-let-7c, hsa-miR-125b-2*	21	+	16364713	hsa-let-7c, hsa-miR-125b-2, hsa-miR-99^b^,^c^	70 nts upstream	1.77
XBP1	hsa-miR-210	11	-	557022	hsa-mir-210	200 nts upstream	1.8
**Predicted repressors:**							
CEBPA	hsa-miR-130a	11	+	57162425	hsa-mir-130a	1500 nts downstream	1.78
TCF2	hsa-miR-31	9	-	21444270	hsa-mir-31	1500 nts upstream	3.12

TSS–transcription start site.

TFBS–transcription factor binding site (predicted).

a -Z score of TF binding site prediction, according to the UCSC browser.

b -hsa-mir-125b which is also derived from this cluster was not co-expressed with CEBPA, however, this miRNA is derived from two different genomic loci, which may mask the expression of hsa-miR-125b from the current cluster (since the other copy is not predicted to be regulated by CEBPA).

c -hsa-mir-99a was not expressed in any of the samples, yet, co-expression of two out of the three members of the cluster, which are different in sequence (and therefore cross-hybridization to the microarray was not likely to have occurred), together with CEBPA gene, supports this prediction.

### The effect of miR-122 overexpression on hESC differentiation

**Figure 5:**
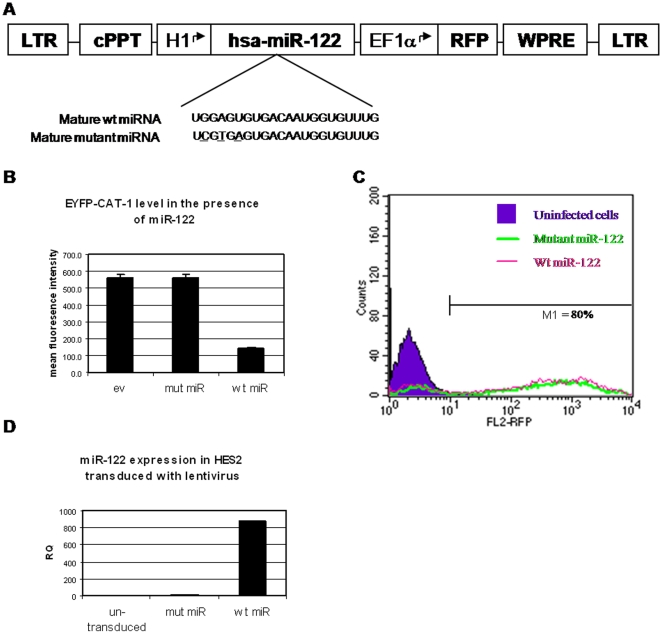
Overexpression of hsa-miR-122 in hESC. A. Schematic representation of the lentiviral vector expressing the wt and mutant hsa-miR-122. The H1 and EF1α promoters are marked by an arrow. The sequence of mature wt miR-122 and mutant miR-122 carrying 3 mutations in the seed sequence (underlined) are shown below. RFP-Red Fluorescent Protein; cPPT-central PolyPurine Tract; WPRE-Woodchuck hepatitis virus Posttranscriptional Regulatory Element. B. HEK-293 cells were co-transfected with a lentiviral plasmid encoding EYFP fused to the 3′UTR of hsa-miR-122 target gene CAT-1 (EYFP-CAT-1) and lentiviral plasmids encoding wt hsa-miR-122, mutant (mut) hsa-miR-122 or empty vector (ev). 48 hr later, the cells were analyzed for EYFP expression by FACS. Each transfection was performed in triplicate and the average mean fluorescence intensity of each triplicate is presented with the SD. C. FACS histogram showing transduction efficiency of HES2 cells with lentiviral vector expressing hsa-miR-122 (wt or mutant) and a RFP reporter. A representative histogram of two independent experiments is shown. D. QRT-PCR results of hsa-miR-122 expression levels in undifferentiated untransduced HES2 cells, or cells transduced with lentiviral vector encoding wt or mutant (mut) hsa-miR-122. Results are expressed as relative quantification of the miRNA expression level in each sample relative to untransduced cells, and normalized to RNU43. Each reaction was performed in triplicate and the average ct was used for RQ calculation.

Some miRNAs may play a role in promoting and maybe even directing differentiation. For example, it was recently shown that overexpression of the brain-specific miR-124 in HeLa cells shifted their gene-expression profile towards that of neurons, whereas delivery of the muscle-specific miR-1 shifted the profile towards that of muscle [50]. In line with these results, we tested whether overexpression of a single miRNA in hESC will affect the differentiation process. As demonstrated above, NaB treatment induced the expression of liver-specific miR-122 in parallel to several hepatic genes, mainly in HES2 cells. miR-122 is highly-expressed in the developing and in the adult liver (Fig. 2 and [31]) and regulates metabolic functions in the adult liver such as lipid metabolism [51] and cholesterol biosynthesis [52]. Therefore, we sought to determine whether overexpression of miR-122 may modify the mRNA profile of HES2 cells towards a ”liver-like” pattern. In order to overexpress miR-122 or a control, which is mutated in 3 nucleotides within the seed sequence, we used a lentiviral transduction-based method, which was shown to enable stable and efficient transgene expression in hESC [25], [34]. We inserted the genomic sequence encoding human hsa-miR-122 into a reporter-containing lentiviral vector, under the constitutive polymerase III promoter H1 (Fig. 5A). We confirmed the functionality of the cloned miR-122 by its ability to repress the expression of an EYFP reporter gene fused to the 3′UTR of a known miR-122 target gene, CAT-1 [31]. Following transfection to HEK-293 cells, wt miR-122 repressed the EYFP levels to 25% of the expression level in the presence of mutant miR-122 or empty vector (Fig. 5B). Transduction efficiency of both lentiviral vectors (encoding wt and mutant miR-122) into HES2 cells was at least 80%, as judged by the expression of the red fluorescent reporter protein (Fig. 5C). QRT-PCR analysis revealed that miR-122 was highly expressed in transduced HES2 cells compared to untransduced cells (Fig. 5D). Apparently, the assay primers did not recognize the mutant miRNA form, probably due to the three mismatches.

**Figure 6:**
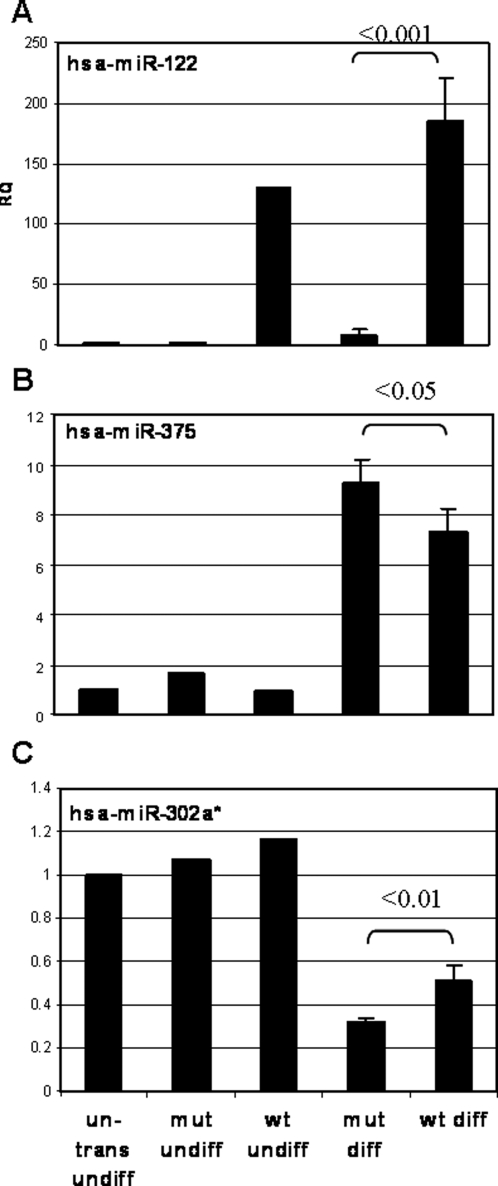
Effect of miR-122 overexpression on miRNA expression in hESC. QRT-PCR results of miRNA expression levels in undifferentiated (undiff) untransduced (un-trans) HES2 cells, and in cells transduced with wt or mutant (mut) miR-122 vector either undifferentiated, or spontaneously differentiated for 14d. Results are expressed as relative quantification of the miRNA expression levels in each sample relative to untransduced cells, and normalized to RNU43. Each reaction was performed in triplicate, and the average ct was used for RQ calculation. For differentiated cells, average RQ of 3 independent experiments is shown with the SD and the p value between mut diff and wt diff cells.

In order to evaluate the effect of miR-122 expression on differentiation of hESC, we transferred the transduced HES2 cells from feeder cells to fibronectin and allowed the cells to differentiate spontaneously in the basic medium without bFGF supplementation for fourteen days. miR-122 remained highly-expressed during the differentiation process (Fig. 6A). We analyzed the global gene expression of differentiated cells expressing either wt or mutant miR-122 using Affymetrix microarrays (Table S2, GEO accession GSE13460 [36]) and qRT-PCR.

**Figure 7:**
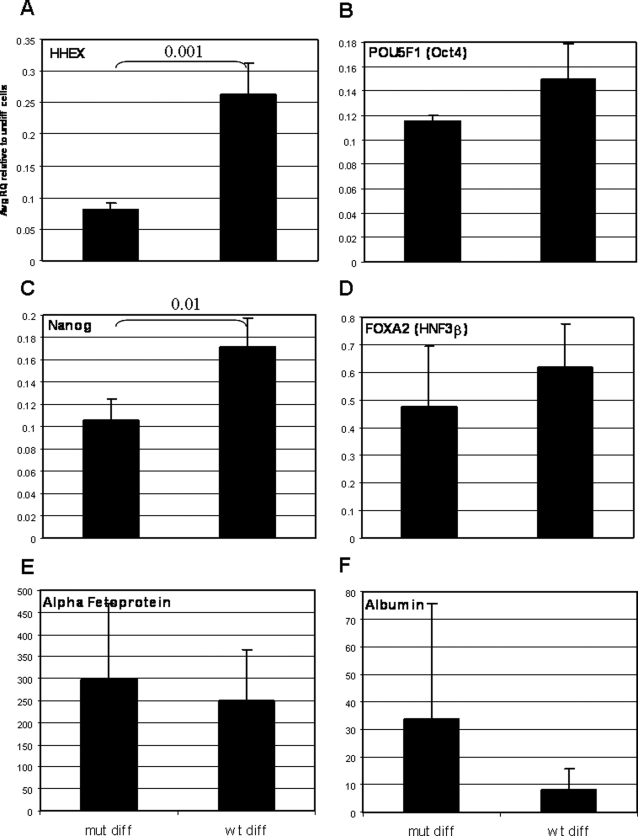
Effect of miR-122 overexpression on gene expression in hESC. QRT-PCR results of gene expression in 14d-differentiated HES2 cells expressing mutant (mut) or wt miR-122. The reactions were performed on the same RNA samples that were analyzed on the microarrays. Results are expressed as relative quantification of the gene level in each sample relative to undifferentiated cells transduced with the relevant miRNA (wt or mutant), and normalized to GUSB. Each reaction was performed in triplicates and the average RQ of 3 independent experiments is shown with the SD and the p value between mut diff and wt diff cells when significant.

Table S3 lists the 50 most differentially-expressed probes between cells transduced with wt versus mutant miR-122. Strikingly, among the genes that were upregulated in the presence of wt miR-122 were HHEX, an early marker of embryonic liver [53], which play a fundamental role in liver development [54] (Table S3 and Fig. 7A), and CXCR4, a marker for definitive endoderm [55]. Interestingly, out of the top 50 upregulated probes, 20% represented markers of undifferentiated hESC, including POU5F1 (Oct4) [56], NANOG [57] and SOX2 [58], which are essential for maintenance of the ES cell pluripotency (Table S3 and Fig. 7B, C). Further, a few hepatic/endoderm markers such as FOXA2, Alpha-fetoprotein, Albumin (Fig. 7D–F) and miR-375 (Fig. 6B) were not upregulated, and there was a concomitant increase in the expression of the ESC-specific miR-302a* (Fig. 6C).

It is noteworthy that only one predicted target of miR-122 (NPEPPS) was significantly downregulated in wt versus mutant miR-122-expressing cells, albeit relatively mildly (1.2-fold). Our interpretation of this observation is that since we profiled gene expression in 14d-differentiated cells stably expressing miR-122, most of the detected changes in gene expression were a result of an indirect rather than direct effect of miR-122.

We looked for enrichment in specific pathways among the significantly differentially-expressed genes when comparing wt versus mutant miR-122-expressing cells (using the Panther classification system http://www.pantherdb.org/). We found one significant enrichment (p<5.46xe^−5^) of the integrin signaling pathway among the downregulated genes. Integrins can activate, among others, the Grb2/Mek pathway [59], which was recently shown to repress Nanog in murine ES cells differentiation towards primitive endoderm [60]. Downregulation of members of the integrin signaling pathway by miR-122 may lead, therefore, to de-repression of Nanog, and consequently, to activation of hESC markers including POU5F1 and SOX2 [61], as was observed in our cells. Additionally, overexpression of miR-122 may have affected global processing of miRNAs (DICER1 is a predicted target of miR-122 according to TargetScan 4.0) and interfered with the ESC differentiation, as it has been previously shown that global loss of small RNAs in Dicer^−/−^ mES cells results in a block in ES cell differentiation [62].

Overall, overexpression of miR-122 alone in hESC was unable to modify the mRNA profile of the cells towards an endodermal or a hepatic pattern, but rather delayed the differentiation when compared to mutant miRNA-expressing cells. Multiple reasons may account for this result: many targets may not be expressed in hESC; some of the targets may only be translationally repressed; it could be that the level of the exogenous miRNA has not reached the level observed in liver as early as seven weeks post-gestation, and some effects may be difficult to detect with the tools and experimental design of our study. Further, the most likely scenario is that miR-122 may require additional miRNAs or proteins in order to allow for differentiation,

In summary, miRNA expression profiling in hESC revealed three novel undiscovered miRNAs, which will be uploaded to the miRbase and given formal names. Upon treatment with NaB, induction of the endodermal miR-122 and miR-375 was observed in parallel to induction of hepatic gene expression, while ESC-specific miRNA expression was reduced. Stable overexpression of endoderm-specific miR-122 in hESC resulted in increased expression of a few endodermal markers in spontaneously-differentiating hESC, but had no clear effect on directing differentiation towards an endodermal fate; rather, it delayed the general differentiation of hESC. Overall, our results demonstrate that miRNA expression correlates with gene expression in differentiated cells, and highlight the potential role of miRNAs in endodermal differentiation of hESC. Additional work is necessary to clarify the specific role of individual miRNAs in endodermal and general hESC differentiation, and its relevance to human embryonic development.

## Supporting Information

Table S1 miroRNA expression in undifferentiated and differentiated hESC
(0.09 MB XLS)

Table S2 Affymetrix gene expression of hESC overexpressing miR-122
(6.72 MB XLS)

Table S3 Effect of miR-122 overexpression on gene expression in differentiated hESC
(0.11 MB DOC)

## References

[1] Hutvagner G, Zamore PD (2002). A microRNA in a multiple-turnover RNAi enzyme complex.. Science.

[2] Llave C, Xie Z, Kasschau KD, Carrington JC (2002). Cleavage of Scarecrow-like mRNA targets directed by a class of Arabidopsis miRNA.. Science.

[3] Mansfield JH, Harfe BD, Nissen R, Obenauer J, Srineel J (2004). MicroRNA-responsive ‘sensor’ transgenes uncover Hox-like and other developmentally regulated patterns of vertebrate microRNA expression.. Nat Genet.

[4] Yekta S, Shih IH, Bartel DP (2004). MicroRNA-directed cleavage of HOXB8 mRNA.. Science.

[5] Olsen PH, Ambros V (1999). The lin-4 regulatory RNA controls developmental timing in Caenorhabditis elegans by blocking LIN-14 protein synthesis after the initiation of translation.. Dev Biol.

[6] Bartel DP (2004). MicroRNAs: genomics, biogenesis, mechanism, and function.. Cell.

[7] Lagos-Quintana M, Rauhut R, Yalcin A, Meyer J, Lendeckel W (2002). Identification of tissue-specific microRNAs from mouse.. Curr Biol.

[8] Wienholds E, Kloosterman WP, Miska E, Alvarez-Saavedra E, Berezikov E (2005). MicroRNA expression in zebrafish embryonic development.. Science.

[9] Alvarez-Garcia I, Miska EA (2005). MicroRNA functions in animal development and human disease.. Development.

[10] Reubinoff BE, Pera MF, Fong CY, Trounson A, Bongso A (2000). Embryonic stem cell lines from human blastocysts: somatic differentiation in vitro.. Nat Biotechnol.

[11] Thomson JA, Itskovitz-Eldor J, Shapiro SS, Waknitz MA, Swiergiel JJ (1998). Embryonic stem cell lines derived from human blastocysts.. Science.

[12] Dvash T, Benvenisty N (2004). Human embryonic stem cells as a model for early human development.. Best Pract Res Clin Obstet Gynaecol.

[13] Houbaviy HB, Murray MF, Sharp PA (2003). Embryonic stem cell-specific MicroRNAs.. Dev Cell.

[14] Tang F, Hajkova P, Barton SC, Lao K, Surani MA (2006). MicroRNA expression profiling of single whole embryonic stem cells.. Nucleic Acids Res.

[15] Strauss WM, Chen C, Lee CT, Ridzon D (2006). Nonrestrictive developmental regulation of microRNA gene expression.. Mamm Genome.

[16] Chen C, Ridzon D, Lee CT, Blake J, Sun Y (2007). Defining embryonic stem cell identity using differentiation-related microRNAs and their potential targets.. Mamm Genome.

[17] Suh MR, Lee Y, Kim JY, Kim SK, Moon SH (2004). Human embryonic stem cells express a unique set of microRNAs.. Dev Biol.

[18] Josephson R, Ording CJ, Liu Y, Shin S, Lakshmipathy U (2007). Qualification of embryonal carcinoma 2102Ep as a reference for human embryonic stem cell research.. Stem Cells.

[19] Lakshmipathy U, Love B, Goff LA, Jornsten R, Graichen R (2007). MicroRNA Expression Pattern of Undifferentiated and Differentiated Human Embryonic Stem Cells.. Stem Cells Dev.

[20] Laurent LC, Chen J, Ulitsky I, Mueller FJ, Lu C (2008). Comprehensive MicroRNA Profiling Reveals a Unique Human Embryonic Stem Cell Signature Dominated by a Single Seed Sequence.. Stem Cells.

[21] Krichevsky AM, Sonntag KC, Isacson O, Kosik KS (2006). Specific microRNAs modulate embryonic stem cell-derived neurogenesis.. Stem Cells.

[22] Tay YM, Tam WL, Ang YS, Gaughwin PM, Yang HH (2007). MicroRNA-134 Modulates the Differentiation of Mouse Embryonic Stem Cells where it Causes Post-transcriptional Attenuation of Nanog and LRH1.. Stem Cells.

[23] Jiang J, Au M, Lu K, Eshpeter A, Korbutt G (2007). Generation of insulin-producing islet-like clusters from human embryonic stem cells.. Stem Cells.

[24] Rambhatla L, Chiu CP, Kundu P, Peng Y, Carpenter MK (2003). Generation of hepatocyte-like cells from human embryonic stem cells.. Cell Transplant.

[25] Ben-Dor I, Itsykson P, Goldenberg D, Galun E, Reubinoff BE (2006). Lentiviral vectors harboring a dual-gene system allow high and homogeneous transgene expression in selected polyclonal human embryonic stem cells.. Mol Ther.

[26] Cohen MA, Itsykson PE, Reubinoff BE (2007). Neural differentiation of human embryonic stem cells.. Current Protocols in Cell Biology.

[27] Barad O, Meiri E, Avniel A, Aharonov R, Barzilai A (2004). MicroRNA expression detected by oligonucleotide microarrays: system establishment and expression profiling in human tissues.. Genome Res.

[28] Bentwich I, Avniel A, Karov Y, Aharonov R, Gilad S (2005). Identification of hundreds of conserved and nonconserved human microRNAs.. Nat Genet.

[29] Katzenellenbogen M, Mizrahi L, Pappo O, Klopstock N, Olam D (2007). Molecular mechanisms of the chemopreventive effect on hepatocellular carcinoma development in Mdr2 knockout mice.. Mol Cancer Ther.

[30] Saini HK, Griffiths-Jones S, Enright AJ (2007). Genomic analysis of human microRNA transcripts.. Proc Natl Acad Sci U S A.

[31] Chang j, Nicolas E, Marks D, Sander C, Lerro A, Buendia MA, Xu C, Mason WS, Moloshok T, Bort R, Zaret KS, Taylor JM (2004). miR-122, a Mammalian Liver-Specific microRNA, is Processed from hcr mRNA and May Downregulate the High Affinity Cationic Amino Acid Transporter CAT-1.. RNA Biology.

[32] Dull T, Zufferey R, Kelly M, Mandel RJ, Nguyen M (1998). A third-generation lentivirus vector with a conditional packaging system.. J Virol.

[33] Zufferey R, Dull T, Mandel RJ, Bukovsky A, Quiroz D (1998). Self-inactivating lentivirus vector for safe and efficient in vivo gene delivery.. J Virol.

[34] Gropp M, Itsykson P, Singer O, Ben-Hur T, Reinhartz E (2003). Stable genetic modification of human embryonic stem cells by lentiviral vectors.. Mol Ther.

[35] Gropp M, Reubinoff BE (2007). Lentiviral-RNA-interference system mediating homogenous and monitored level of gene silencing in human embryonic stem cells.. Cloning Stem Cells.

[36] Kosaka M, Nishina Y, Takeda M, Matsumoto K, Nishimune Y (1991). Reversible effects of sodium butyrate on the differentiation of F9 embryonal carcinoma cells.. Exp Cell Res.

[37] Osafune K, Caron L, Borowiak M, Martinez RJ, Fitz-Gerald CS (2008). Marked differences in differentiation propensity among human embryonic stem cell lines.. Nat Biotechnol.

[38] Fukuda Y, Kawasaki H, Taira K (2005). Exploration of human miRNA target genes in neuronal differentiation.. Nucleic Acids Symp Ser (Oxf).

[39] Kikuchi Y, Verkade H, Reiter JF, Kim CH, Chitnis AB (2004). Notch signaling can regulate endoderm formation in zebrafish.. Dev Dyn.

[40] Garzon R, Pichiorri F, Palumbo T, Iuliano R, Cimmino A (2006). MicroRNA fingerprints during human megakaryocytopoiesis.. Proc Natl Acad Sci U S A.

[41] Martinez-Ceballos E, Chambon P, Gudas LJ (2005). Differences in gene expression between wild type and Hoxa1 knockout embryonic stem cells after retinoic acid treatment or leukemia inhibitory factor (LIF) removal.. J Biol Chem.

[42] Viswanathan SR, Daley GQ, Gregory RI (2008). Selective blockade of microRNA processing by Lin28.. Science.

[43] Poy MN, Eliasson L, Krutzfeldt J, Kuwajima S, Ma X (2004). A pancreatic islet-specific microRNA regulates insulin secretion.. Nature.

[44] Landgraf P, Rusu M, Sheridan R, Sewer A, Iovino N (2007). A mammalian microRNA expression atlas based on small RNA library sequencing.. Cell.

[45] Mohana Kumar B, Song HJ, Cho SK, Balasubramanian S, Choe SY (2007). Effect of histone acetylation modification with sodium butyrate, a histone deacetylase inhibitor, on cell cycle, apoptosis, ploidy and gene expression in porcine fetal fibroblasts.. J Reprod Dev.

[46] Tanimizu N, Nishikawa M, Saito H, Tsujimura T, Miyajima A (2003). Isolation of hepatoblasts based on the expression of Dlk/Pref-1.. J Cell Sci.

[47] Fuchs O (2007). Growth-inhibiting activity of transcription factor C/EBPalpha, its role in haematopoiesis and its tumour suppressor or oncogenic properties in leukaemias.. Folia Biol (Praha).

[48] Lee YS, Dutta A (2007). The tumor suppressor microRNA let-7 represses the HMGA2 oncogene.. Genes Dev.

[49] Mizuno Y, Yagi K, Tokuzawa Y, Kanesaki-Yatsuka Y, Suda T (2008). miR-125b inhibits osteoblastic differentiation by down-regulation of cell proliferation.. Biochem Biophys Res Commun.

[50] Lim LP, Lau NC, Garrett-Engele P, Grimson A, Schelter JM (2005). Microarray analysis shows that some microRNAs downregulate large numbers of target mRNAs.. Nature.

[51] Esau C, Davis S, Murray SF, Yu XX, Pandey SK (2006). miR-122 regulation of lipid metabolism revealed by in vivo antisense targeting.. Cell Metab.

[52] Krutzfeldt J, Rajewsky N, Braich R, Rajeev KG, Tuschl T (2005). Silencing of microRNAs in vivo with ‘antagomirs’.. Nature.

[53] Xanthopoulos KG, Mirkovitch J (1993). Gene regulation in rodent hepatocytes during development, differentiation and disease.. Eur J Biochem.

[54] Hunter MP, Wilson CM, Jiang X, Cong R, Vasavada H (2007). The homeobox gene Hhex is essential for proper hepatoblast differentiation and bile duct morphogenesis.. Dev Biol.

[55] D'Amour KA, Agulnick AD, Eliazer S, Kelly OG, Kroon E (2005). Efficient differentiation of human embryonic stem cells to definitive endoderm.. Nat Biotechnol.

[56] Niwa H, Miyazaki J, Smith AG (2000). Quantitative expression of Oct-3/4 defines differentiation, dedifferentiation or self-renewal of ES cells.. Nat Genet.

[57] Chambers I, Colby D, Robertson M, Nichols J, Lee S (2003). Functional expression cloning of Nanog, a pluripotency sustaining factor in embryonic stem cells.. Cell.

[58] Avilion AA, Nicolis SK, Pevny LH, Perez L, Vivian N (2003). Multipotent cell lineages in early mouse development depend on SOX2 function.. Genes Dev.

[59] Clark EA, Brugge JS (1995). Integrins and signal transduction pathways: the road taken.. Science.

[60] Hamazaki T, Kehoe SM, Nakano T, Terada N (2006). The Grb2/Mek pathway represses Nanog in murine embryonic stem cells.. Mol Cell Biol.

[61] Boyer LA, Lee TI, Cole MF, Johnstone SE, Levine SS (2005). Core transcriptional regulatory circuitry in human embryonic stem cells.. Cell.

[62] Kanellopoulou C, Muljo SA, Kung AL, Ganesan S, Drapkin R (2005). Dicer-deficient mouse embryonic stem cells are defective in differentiation and centromeric silencing.. Genes Dev.

