# Supplementary material for: MicroRNA Expression Patterns and Function in Endodermal Differentiation of Human Embryonic Stem Cells
Source: PLoS One. 2008 Nov 18;3(11):e3726. doi: 10.1371/journal.pone.0003726 (PMC2581805; doi:10.1371/journal.pone.0003726)
Supplement: Table S3 — Effect of miR-122 overexpression on gene expression in differentiated hESC (0.11 MB DOC) [file pone.0003726.s003.doc]

**Table S**3: Effect of miR-122 overexpression on gene expression in differentiated hESC

| Top 50 probes upregulated or downregulated in wt versus mutant miR-122-expressing cells | | | | | |
| --- | --- | --- | --- | --- | --- |
| **Upregulated probes** | | | **Downregulated probes** | | |
| **Gene Symbol** | **Exp diff** | ***P* value** | **Gene Symbol** | **Exp diff** | ***P* value** |
| ALPPL2 | 2.65 | 0.0319 | HSD3B1 | 0.34 | 0.0185 |
| MT1M | 2.22 | 0.0129 | PLAC8 | 0.38 | 0.0490 |
| **PTPRZ1** | 2.21 | 0.0019 | CXCR7 | 0.44 | 0.0416 |
| MMP1 | 2.13 | 0.0191 | CGA | 0.45 | 0.0108 |
| MT1G | 2.07 | 0.0181 | CRH | 0.47 | 0.0406 |
| MT1E | 2.03 | 0.0115 | TGM2 | 0.48 | 0.0078 |
| MT1H | 2.01 | 0.0152 | VTCN1 | 0.50 | 0.0277 |
| TRPC6 | 2.00 | 0.0111 | DKK2 | 0.51 | 0.0448 |
| ALPP /// ALPPL2 | 1.98 | 0.0204 | HAND1 | 0.51 | 0.0028 |
| MT1X | 1.98 | 0.0194 | SYNPO | 0.53 | 0.0142 |
| HHEX | 1.97 | 0.0010 | PTN | 0.53 | 0.0005 |
| MT1M | 1.95 | 0.0200 | LUM | 0.53 | 0.0206 |
| C6orf148 | 1.89 | 0.0015 | IGFBP3 | 0.53 | 0.0144 |
| MT1F | 1.88 | 0.0127 | OLFML2B | 0.54 | 0.0477 |
| MT1X | 1.88 | 0.0365 | SMARCA2 | 0.55 | 0.0014 |
| HHEX | 1.88 | 0.0194 | COL5A1 | 0.55 | 0.0270 |
| MT1F | 1.87 | 0.0150 | MFAP5 | 0.55 | 0.0062 |
| DEPDC6 | 1.86 | 0.0289 | GABRP | 0.55 | 0.0210 |
| **TDGF1 /// TDGF3** | 1.85 | 0.0133 | PTN | 0.55 | 0.0420 |
| MT2A | 1.82 | 0.0262 | EPAS1 | 0.56 | 0.0087 |
| **DAZL** | 1.79 | 0.0246 | LRRN3 | 0.56 | 0.0113 |
| LOC645745 | 1.78 | 0.0086 | COL3A1 | 0.56 | 0.0019 |
| LRP8 | 1.77 | 0.0230 | AQP1 | 0.56 | 0.0113 |
| NGFR | 1.75 | 0.0394 | MBD2 | 0.56 | 0.0484 |
| **NANOG** | 1.73 | 0.0223 | CDKN2B | 0.57 | 0.0264 |
| GNAS | 1.71 | 0.0398 | COL5A1 | 0.57 | 0.0091 |
| CKMT1B /// CKMT1A | 1.71 | 0.0012 | GPC5 | 0.57 | 0.0430 |
| **UTF1** | 1.69 | 0.0098 | COL15A1 | 0.58 | 0.0337 |
| FZD5 | 1.68 | 0.0079 | IL1R1 | 0.59 | 0.0199 |
| FOXG1B | 1.68 | 0.0312 | COL5A1 | 0.59 | 0.0343 |
| PYCARD | 1.68 | 0.0064 | CHN2 | 0.59 | 0.0014 |
| **POLR3G** | 1.67 | 0.0098 | SLC1A4 | 0.59 | 0.0200 |
| **DNMT3B** | 1.65 | 0.0015 | IGFBP3 | 0.59 | 0.0287 |
| FA2H | 1.65 | 0.0241 | MS4A6A | 0.59 | 0.0032 |
| NFIB | 1.64 | 0.0054 | ANKS1A | 0.59 | 0.0351 |
| GPR64 | 1.62 | 0.0008 | EPB41L3 | 0.59 | 0.0012 |
| TTYH1 | 1.62 | 0.0029 | STS | 0.60 | 0.0399 |
| **JARID2** | 1.61 | 0.0030 | IGFBP7 | 0.60 | 0.0006 |
| ITGB1BP3 | 1.61 | 0.0102 | SLC27A6 | 0.60 | 0.0198 |
| ACTN3 | 1.61 | 0.0078 | NRP1 | 0.60 | 0.0335 |
| NP | 1.59 | 0.0012 | DSCR1 | 0.60 | 0.0404 |
| CXCR4 | 1.57 | 0.0476 | IQWD1 | 0.60 | 0.0397 |
| **ZIC3** | 1.56 | 0.0012 | AQP1 | 0.60 | 0.0149 |
| UGT8 | 1.56 | 0.0209 | GUCY1A3 | 0.60 | 0.0447 |
| **SOX2** | 1.56 | 0.0370 | COL3A1 | 0.60 | 0.0121 |
| LOC642559 | 1.55 | 0.0225 | IGFBP7 | 0.61 | 0.0469 |
| RABGAP1L | 1.55 | 0.0439 | HTRA1 | 0.61 | 0.0045 |
| **POU5F1 /// POU5F1P1** | 1.54 | 0.0078 | PRSS12 | 0.61 | 0.0398 |
| RASGRP2 | 1.53 | 0.0051 | FAT4 | 0.61 | 0.0403 |
| CHODL | 1.51 | 0.0044 | PIK3R1 | 0.61 | 0.0038 |

The top 50 probes significantly (p<0.05) upregulated or downregulated in 14d-spontaneously differentiated wt compared to mutant miR-122-expressing cells. Average values calculated from 3 (mutant miRNA) or 2 (wt miRNA) independent experiments were used for the analysis. A gene is shown more than once in either list in case that more than one of its probes has fulfilled the above criteria. ESC markers are **bolded**; Exp diff – ratio of wt to mutant miR-122-expressing cells gene expression value.
